# Magnetic zero-modes, vortices and Cartan geometry

**DOI:** 10.1007/s11005-017-1023-2

**Published:** 2017-11-07

**Authors:** Calum Ross, Bernd J. Schroers

**Affiliations:** 1 Department of Mathematics, Heriot-Watt University, Edinburgh, EH14 4AS UK; 2 Maxwell Institute for Mathematical Sciences, Edinburgh, UK

**Keywords:** Magnetic zero-modes, Dirac operator, Vortex equations, Cartan geometry, 53Z05, 58Z05

## Abstract

We exhibit a close relation between vortex configurations on the 2-sphere and magnetic zero-modes of the Dirac operator on $\mathbb {R}^3$ which obey an additional nonlinear equation. We show that both are best understood in terms of the geometry induced on the 3-sphere via pull-back of the round geometry with bundle maps of the Hopf fibration. We use this viewpoint to deduce a manifestly smooth formula for square-integrable magnetic zero-modes in terms of two homogeneous polynomials in two complex variables.

## Introduction

The goal of this paper is to explain and exploit a link between magnetic zero-modes of the Dirac operator on Euclidean 3-space and vortices on the 2-sphere via a particular family of geometries on the 3-sphere.

The 3-sphere geometries are obtained from the standard round geometry via pull-back with a family of maps $S^3\rightarrow S^3$ which are bundle maps of the Hopf fibration and cover holomorphic maps $S^2\rightarrow S^2$. The bundle maps are given in terms of two complex polynomials, and one consequence of our analysis is a manifestly smooth and square-integrable expression both for the magnetic zero-modes and the vortex configurations in terms of these polynomials. Another is an interpretation of vortices on $S^2$ in terms of Cartan geometry. In the remainder of this introduction we sketch the context for our results.

The problem of determining magnetic zero-modes of the Dirac operator in Euclidean 3-space was first posed and addressed in an influential paper by Loss and Yau [1] in 1986. Motivated by questions about the stability of atoms, the authors were interested in finding spinors $\varPsi$ and magnetic gauge potentials *A* on $\mathbb {R}^3$ such that $\varPsi$ is a zero-mode of the (static) Dirac operator minimally coupled to *A*, and both the associated magnetic field and the spinor are square-integrable. In this paper, we call pairs of spinors $\varPsi$ and magnetic gauge potentials *A* satisfying this condition magnetic zero-modes.

Loss and Yau gave explicit expressions for one family of magnetic zero-modes, which we call linear in the following, and derived a formula which determines a gauge field for a given spinor field such that the pair form a magnetic zero-mode. This formula is singular where the spinor field vanishes, but it was, nonetheless, used by Adam, Muratori and Nash (AMN) in a series of papers [2–4] to obtain magnetic zero-modes which satisfy an additional nonlinear equation, and which we call vortex zero-modes in this paper. AMN observed that their solutions can be expressed in terms of solutions of the Liouville equation on $S^2$ and addressed the singularities in the resulting formulae. In [2], they also pointed out that the coupled Dirac and nonlinear equation can be obtained as the dimensional reduction in a perturbed Seiberg–Witten equation on $\mathbb {R}^4$ with a crucial sign flip (the resulting equation is often called the Freund equation).

In 2000, Erdös and Solovej pointed out that the geometry underlying the existence of magnetic zero-modes is the conformal equivalence of $\mathbb {R}^3$ and $S^3{\setminus } \{ \text {point}\}$ and the Hopf fibration of $S^3$ over $S^2$ [5]. This was used in [6, 7] to show that the linear magnetic zero-modes found by Loss and Yau can be obtained directly by pulling eigenmodes (of any energy) of the Dirac operator on $S^3$ back to $\mathbb {R}^3$.

One motivation for this paper was to find a similarly geometrical but also explicit understanding of the vortex zero-modes, i.e. to use the geometrical insight of Erdös and Solovej for a better understanding and improvement in the formulae derived by AMN. A second motivation was to explore links to a vortex equation for a scalar Higgs field and an abelian gauge field on $S^2$, recently proposed by Popov. The existence of such links is suggested by the appearance of the same data in Popov vortex solutions and the AMN expressions for magnetic zero-modes; it is the reason why we call the latter vortex zero-modes.

The Popov vortex equations were obtained in [8] as the reduction by *SU*(1, 1) symmetry of the self-duality equations for *SU*(1, 1) Yang–Mills theory on the product of the 2-sphere with hyperbolic 2-space. In [9], Manton pointed out that the Popov vortex equations can be solved in terms of rational maps $S^2\rightarrow S^2$. His solution turns out to be a particularly simple illustration of an interesting subsequent observation by Baptista [10] that Bogmol’nyi vortex equations on a Kähler surface can be interpreted as degenerate Hermitian metrics.

In the terminology of Baptista’s paper, Manton showed that Popov vortices encode the geometry of the pull-back of the round metric on $S^2$ with a rational map. If the rational map has degree *n*, the metric necessarily has conical singularities at the $2n-2$ ramification points, which are also the zeros of the vortex Higgs field.

Here, we lift this picture from $S^2$ to $S^3$. This is geometrically natural for Popov vortices, since they live on a *U*(1) bundle over $S^2$ whose total space is the Lens space $S^3/\mathbb {Z}_{2n-2}$. Manton’s rational map characterising the vortex lifts to a bundle map $S^3\rightarrow S^3$, and the pull-back of the round metric on $S^3$ with this bundle map defines a metric which, generalising Baptista’s viewpoint, encodes a vortex configuration on $S^3$. We then show that such a vortex configuration defines a magnetic zero-mode of the Dirac operator on $S^3$. Using conformal equivalence we obtain the advertised smooth and manifestly square-integrable expression for vortex zero-modes on $\mathbb {R}^3$ and, at the same time, establish the expected link to Popov vortices.

The fact that $S^3$ is the Lie group *SU*(2) allows one to encode the round geometry of $S^3$ in the Maurer–Cartan form $h^{-1}\hbox {d}h$. In Cartan geometry, the same form also encodes the geometry of the round geometry of $S^2$ by combining the orthonormal frame field with the spin connection 1-form of $S^2$. Since all the geometries we discuss in this paper are pulled back from the round geometries of $S^2$ and $S^3$, it is not surprising that many of our results can be stated succinctly in terms of the pull-back of the Maurer–Cartan form via the bundle map $S^3\rightarrow S^3$. In fact, the flatness condition of the *su*(2) gauge potentials defined by these pull-backs turns out to be equivalent to our vortex equations on $S^3$ and to the Popov vortex equations on $S^2$. This adds a further, non-abelian interpretation of the vortex zero-modes. It also provides an intriguing link with the self-duality equations for *SU*(1, 1) gauge fields from which the Popov equations arose.

In this paper we are interested in the geometry linking magnetic zero-modes and vortices, but also in manifestly smooth expressions for both. The paper contains a number of explicit calculations and formulae, and we therefore need to lay out conventions and coordinates in some detail at the beginning. To help the reader keep sight of the bigger picture, we have also produced a summary diagram of the geometries and the maps between them in Fig. 3. Although the picture is part of our final summary section, the reader may find it helpful to refer to it now or while reading the paper.

The paper is organised as follows. In Sect. 2 we collect our conventions for parameterising *SU*(2) both as a Lie group and a round 3-sphere, give the stereographic and gnomonic projection from $S^3$ and $\mathbb {R}^3$ in these coordinates, and explain how both enter a simple formula for relating orthonormal frames on $S^3$ and $\mathbb {R}^3$ and for mapping zero-modes of magnetic Dirac operators on $S^3$ to zero-modes of magnetic Dirac operators on $\mathbb {R}^3$. While the conformal covariance of the kernel of the Dirac operator is, of course, well known, we are not aware of a treatment which emphasises the role of the gnomonic projection in the way we do. We illustrate our discussion by constructing the linear magnetic zero-modes on $\mathbb {R}^3$ from general eigenmodes on $S^3$ in our conventions.

Section 3 contains our definition of vortex configurations on $S^3$ and some of our main results: the equivalence between vortex configurations on $S^3$ and flat *su*(2) gauge potentials, an expression for both in terms of bundle maps $S^3\rightarrow S^3$, and the construction of magnetic zero-modes on both $S^3$ and $\mathbb {R}^3$ from vortex configurations. The allowed bundle maps can be expressed in terms of two polynomials, thus leading to the promised formulae for magnetic zero-modes. The section ends with a brief discussion of how linear and vortex zero-modes can be combined to form new magnetic zero-modes.

In Sect. 4 we review the definition of Popov vortices and show that our vortex configurations on $S^3$ are equivariant descriptions of them. We explain the relation between our bundle map $S^3\rightarrow S^3$ and the rational map $S^2\rightarrow S^2$ used by Manton for solving the Popov equations and interpret the pull-back to $S^2$ of the flat *su*(2) gauge potential on $S^3$ in the language of Cartan geometry.

Finally, Sect. 5 contains a summary in the form of a diagram in Fig. 3 and an outlook onto open questions.

## Magnetic Dirac operators on $S^3$ and on $\mathbb {R}^3$

### Conventions for *SU*(2) and the Hopf map

We adopt the conventions of [11, 12] and use the *su*(2) generators

2.1 $$\begin{aligned} t_j=-\frac{i}{2}\tau _j, \quad j=1,2,3, \end{aligned}$$

where the $\tau _j$ are the Pauli matrices, with commutators $[t_i,t_j]=\epsilon _{ijk}t_k$. Often we will work in terms of

2.2 $$\begin{aligned} t_+=t_1+it_2, \quad t_-=t_1-it_2 \end{aligned}$$

with commutators

2.3 $$\begin{aligned} {[}t_3,t_+]=-i t_+, \quad [t_3,t_-]=it_-, \quad [t_+,t_-]=-2it_3. \end{aligned}$$

We parameterise an *SU*(2) matrix *h* in terms of a pair of complex numbers $(z_1,z_2)$ via

2.4 $$\begin{aligned} h= \begin{pmatrix} z_1 &{}\quad -\bar{z}_2\\ z_2 &{}\quad \bar{z}_1 \end{pmatrix}, \end{aligned}$$

with the constraint $$ |z_1|^2 + |z_2|^2= 1$$ understood. The (real) left-invariant 1-forms are defined via

2.5 $$\begin{aligned} h^{-1}\hbox {d}h = \sigma _1 t_1 + \sigma _2t_2 + \sigma _3 t_3 \end{aligned}$$

and satisfy $\hbox {d}\sigma _1 = - \sigma _2 \wedge \sigma _3$ and the cyclic permutations of this equation. Defining

2.6 $$\begin{aligned} \sigma = \sigma _1+i\sigma _2, \quad \bar{\sigma } = \sigma _1-i\sigma _2 \end{aligned}$$

we also note that

2.7 $$\begin{aligned} \hbox {d}\sigma = i\sigma \wedge \sigma _3, \quad \hbox {d}\sigma _3= \frac{i}{2} \bar{\sigma }\wedge \sigma \end{aligned}$$

and have the following expressions in terms of complex coordinates:

2.8 $$\begin{aligned} \sigma = 2i (z_1\hbox {d}z_2 -z_2 \hbox {d}z_1), \quad \sigma _3= 2i (\bar{z}_1\hbox {d}z_1 + \bar{z}_2 \hbox {d}z_2). \end{aligned}$$

The dual vector fields $X_j$, $j=1,2,3$ generate the right action $h\mapsto ht_j$ [11]. Their commutators are

2.9 $$\begin{aligned} {[}X_i,X_j]=\epsilon _{ijk}X_k, \end{aligned}$$

so that, in terms of

2.10 $$\begin{aligned} X_+ = X_1 +iX_2, \quad X_-=X_1 -iX_2, \end{aligned}$$

we have

2.11 $$\begin{aligned} {[}X_+,X_- ]=-2iX_3, \quad [iX_3,X_\pm ]=\pm X_\pm . \end{aligned}$$

In terms of complex coordinates, the right generators are

2.12 $$\begin{aligned} X_+&=i (z_1\bar{\partial }_2 -z_2 \bar{\partial }_1), \nonumber \\ X_-&=i (\bar{z}_2\partial _1 -\bar{z}_1 \partial _2), \nonumber \\ X_{3\;}&=\frac{i}{2} ( \bar{z}_1\bar{\partial }_1 +\bar{z}_2 \bar{\partial }_2 - z_1 \partial _1 -z_2 \partial _2). \end{aligned}$$

The corresponding generators of the left action $h\mapsto -t_jh$ are denoted $Z_\pm$ and $Z_3$; expressions in terms of our complex coordinates are given in [11]. We also note that the Laplace operator on $S^3$ (with radius 2) can be written as

2.13 $$\begin{aligned} \varDelta _{S^3}=X_1^2 +X_2^2 +X_3^2= X_+X_- +iX_3 +X_3^2 = X_-X_+ - iX_3 +X_3^2, \end{aligned}$$

with an analogous expression in terms of $Z_j$. Finally, we have the pairings

2.14 $$\begin{aligned} \bar{\sigma }(X_+)= \sigma (X_-)=2, \quad \sigma _3(X_3)=1, \end{aligned}$$

with all other pairings vanishing.

In this paper we parameterise the 2-sphere via stereographic projection in terms of a coordinate $z\in \mathbb {C}$. We work in coordinates provided by stereographic projection from the south pole, referring to [11] for details and the coordinate changes required to cover the south pole itself by projecting from the north pole. In terms of our complex coordinates (2.4) for $S^3$, the Hopf map is

2.15 $$\begin{aligned} \pi : S^3 \rightarrow S^2, \quad h \mapsto z= \frac{z_2}{z_1}. \end{aligned}$$

Then,

2.16 $$\begin{aligned} s: \mathbb {C}\rightarrow SU(2), \quad z\mapsto \frac{1}{\sqrt{1+|z|^2}}\begin{pmatrix} 1 &{}\quad -\bar{z} \\ z &{}\quad 1 \end{pmatrix} \end{aligned}$$

is a local section of the Hopf bundle. We will use it in this paper to switch between the equivariant description of sections of associated line bundles to local expressions for such sections. Defining spaces of equivariant functions

2.17 $$\begin{aligned} C^\infty (S^3,\mathbb {C})_N= \left\{ F: S^3 \rightarrow \mathbb {C}|2iX_3F=NF\right\} , \quad N\in \mathbb {N}^0 \end{aligned}$$

and writing *H* for the hyperplane bundle over $S^2$, one has the following commutative diagram [11, 12]:2.18


### Stereographic projection and frames

In the remainder of this paper, we consider a 2-sphere of radius $\lambda$ and a 3-sphere of radius $\ell$. Then,

2.19 $$\begin{aligned} \left( \frac{\ell }{2} \sigma _1, \frac{\ell }{2} \sigma _2, \frac{\ell }{2} \sigma _3\right) \end{aligned}$$

provides a convenient orthonormal frame for $S^3$, and the metric is

2.20 $$\begin{aligned} \hbox {d}s^2= \frac{\ell ^2}{4}\left( \sigma _1^2 +\sigma _2^2 +\sigma _3^2\right) . \end{aligned}$$

In order to make contact with the usual orientation on $\mathbb {R}^3$ after stereographic projection, we define the orientation on $S^3$ in terms of the volume element

2.21 $$\begin{aligned} \varOmega = \frac{\ell ^3}{8}\sigma _2\wedge \sigma _1\wedge \sigma _3. \end{aligned}$$

We write elements of $\mathbb {R}^3$ as $\mathbf {x} = (x_1,x_2,x_3)^t$ denote their length by $r=|\mathbf {x}|$, and assume that

2.22 $$\begin{aligned} \left( \hbox {d}x_1, \hbox {d}x_2, \hbox {d}x_3\right) \end{aligned}$$

is an oriented orthonormal frame, so the metric and volume element are

2.23 $$\begin{aligned} \hbox {d}s^2 =\hbox {d}x_1^2 +\hbox {d}x_2^2 +\hbox {d}x_3^2, \quad \hbox {d}x_1\wedge \hbox {d}x_2 \wedge \hbox {d}x_3. \end{aligned}$$

Thinking of the 3-sphere of radius $\ell$ embedded in $\mathbb {R}^4$ with coordinates $(y_1,y_2,y_3,y_4)$, the stereographic projection from the south pole onto $\mathbb {R}^3$ is

2.24 $$\begin{aligned}&\mathrm {St}: S^3{\setminus }\{\mathrm {south \;pole}\} \rightarrow \mathbb {R}^3, \nonumber \\&\quad (y_1,y_2,y_3,y_4)\mapsto (x_1,x_2,x_3)= \left( \frac{\ell y_{1}}{\ell +y_{4}},\frac{\ell y_{2}}{\ell +y_{4}},\frac{\ell y_{3}}{\ell +y_{4}}\right) ,\qquad \end{aligned}$$

with inverse

2.25 $$\begin{aligned}&\mathrm {St}^{-1}:\mathbb {R}^3 \rightarrow S^3, \nonumber \\&\quad (x_1,x_2,x_3)\mapsto (y_1,y_2,y_3,y_4) = \frac{\ell }{\ell ^2+r^2} (2x_1\ell ,2x_2\ell , 2x_3\ell , \ell ^2-r^2).\qquad \quad \end{aligned}$$

Writing $\varvec{\tau } =(\tau _1,\tau _2,\tau _3)$ for the vector whose components are the Pauli matrices, and identifying $(y_1,y_2,y_3,y_4) \in S^3$ with the unitary matrix $(y_4\mathbb {I} + i y_1\tau _1 + i y_2\tau _2+ i y_3\tau _3)/\ell$, the inverse stereographic projection is, up to scale, the map

2.26 $$\begin{aligned}&H:\mathbb {R}^3 \rightarrow SU(2), \nonumber \\&\quad \mathbf {x} \mapsto \frac{\ell ^2-r^2}{\ell ^2 +r^2} \mathbb {I}+\frac{ 2i\ell }{\ell ^2+r^ 2} \mathbf {x} \cdot \varvec{\tau } = \frac{1 }{\ell ^2 +r^2} \begin{pmatrix} {\ell ^2-r^2} +2i\ell x_3 &{}\quad 2i\ell (x_1-ix_2) \\ 2i\ell (x_1+ix_2) &{} \quad {\ell ^2-r^2} -2i\ell x_3 \end{pmatrix}, \end{aligned}$$

so that the Hopf projection in stereographic coordinates is

2.27 $$\begin{aligned} \pi \circ H: (x_1,x_2,x_3) \mapsto \frac{2\ell (x_1+ix_2)}{2x_3\ell + i (r^2-\ell ^2)}. \end{aligned}$$

In the following we will often need to pull back functions, spinors or forms on the 3-sphere of radius $\ell$ to $\mathbb {R}^3$ with the inverse stereographic projection. To simplify notation we will write the pull-back in terms of *H* rather than $\mathrm {St}^{-1}$, even though the two maps strictly speaking take values on 3-spheres of different radii.

A recurring theme in this paper is the interplay between the stereographic projection and the gnomonic projection, often used in cartography, which maps great circles to straight lines. The inverse of the gnomonic projection is the map

**Fig. 1:**
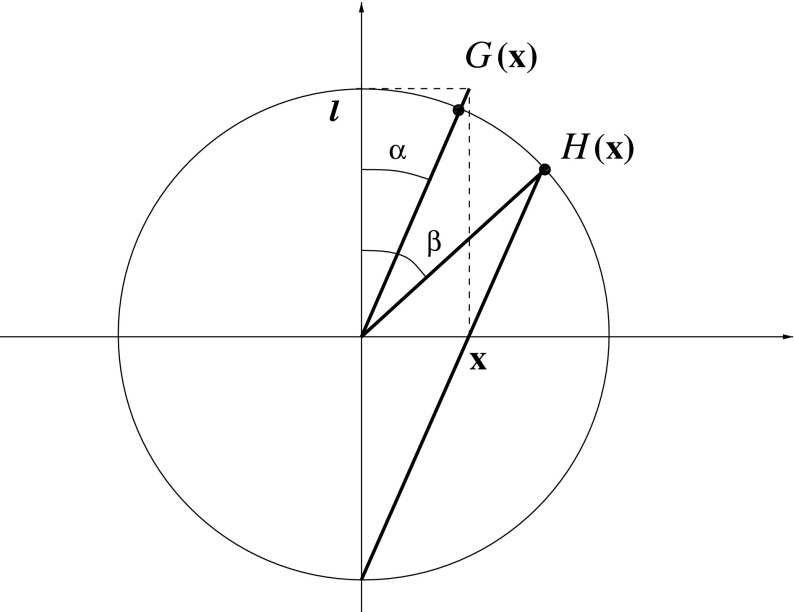
Geometry of the relation between the gnomonic and stereographic projections $G,H: \mathbb {R}^3\rightarrow S^3$ defined in the main text. The elementary relation $\beta = 2\alpha$ implies $H(\mathbf {x})= G^2(\mathbf {x})$

2.28 $$\begin{aligned} G :\mathbb {R}^3\rightarrow SU(2), \quad \mathbf {x} \mapsto \frac{\ell \, \mathbb {I}+ i\mathbf {x}\cdot \varvec{\tau }}{\sqrt{\ell ^2 + r^2}}, \end{aligned}$$

whose image satisfies $G(\mathbf {x})^2=H(\mathbf {x})$. This relation follows immediately from the explicit forms of the maps, but it also follows from the geometric meaning of *G* and *H*, which is illustrated in Fig. 1. For fixed $\mathbf {x}$, $G(\mathbf {x})$ and $H(\mathbf {x})$ are rotations about the same axis, and it follows from elementary geometry that *G* rotates by twice the rotation angle of *H*. As an aside we note that describing spherical geometry in terms of $\mathbb {R}^3$ via pull-back with *H* and *G* is analogous to describing hyperbolic geometry in terms of, respectively, the Poincaré and the Beltrami–Klein models.

The maps *G* and *H* can be used to pull back the left-invariant 1-forms $\sigma _j$, $j=1,2,3$, on the 3-sphere. The relation of the resulting frames on $\mathbb {R}^3$ to each other and to the standard frame (2.22) is interesting, and important for the remainder of this paper. We therefore collect the relevant results here. Defining the scale function

2.29 $$\begin{aligned} \varOmega =\frac{\ell ^2 +r^2}{4\ell }, \end{aligned}$$

we introduce 1-forms $\theta _j$, $j=1,2,3$, on $\mathbb {R}^3$ via

2.30 $$\begin{aligned} H^{-1} \hbox {d}H =\frac{i}{2\varOmega } \varvec{\theta }\cdot \varvec{\tau }, \quad \text {or} \quad \theta _j = - \varOmega H^*\sigma _j. \end{aligned}$$

Then, we find

2.31 $$\begin{aligned} \varvec{\theta } \cdot \varvec{\tau }&= \frac{1}{\ell ^2 + r^2} \left( 2(\mathbf {x}\cdot \varvec{\tau })( \mathbf {x}\cdot \hbox {d} \mathbf {x}) + (\ell ^2 -r^2) \varvec{\tau }\cdot \hbox {d}\mathbf {x} + 2\ell (\mathbf {x}\times \hbox {d}\mathbf {x}) \cdot \varvec{\tau } \right) \nonumber \\&= G^{-1} ( \hbox {d} \mathbf {x}\cdot \varvec{\tau }) G. \end{aligned}$$

In other words, pulling back the 1-forms $\sigma _1,\sigma _2$ and $\sigma _3$ via *H* gives a frame which is related to the standard frame (2.22) by rotation with *G* (acting in its adjoint representation), a reflection in the origin and rescaling by $\varOmega$. For later use we note

2.32 $$\begin{aligned} \theta _3= \frac{1}{\ell ^{2}+r^2}\left( 2(x_1x_3-\ell x_2)\hbox {d}x_1 + 2(x_2x_3+\ell x_1)\hbox {d}x_2 + (\ell ^{2}-r^{2}+2x_3^{2})\hbox {d}x_3\right) . \end{aligned}$$

#### Lemma 2.1

The pull-backs $G^{-1}\hbox {d}G$ and $H^{-1}\hbox {d}H$ of the Maurer–Cartan form on *SU*(2) are related via

2.33 $$\begin{aligned} H^{-1}\hbox {d}H = G^{-1}\hbox {d}G + G^{-1}( G^{-1} \hbox {d}G)G , \end{aligned}$$

and the inverse relation can be expressed as

2.34 $$\begin{aligned} G^{-1} \hbox {d}G = \frac{1}{2} H^{-1}\hbox {d}H + \star ( \hbox {d}\varOmega \wedge H^{-1} \hbox {d}H). \end{aligned}$$

#### Proof

Formula (2.33) is an immediate consequence of $H=G^2$. To show the inverse relation, we use (2.33) to write (2.34) as

2.35 $$\begin{aligned} \star ( 2\hbox {d}\varOmega \wedge (\hbox {d}G G^{-1} + G^{-1} \hbox {d}G))=\hbox {d}G G^{-1} - G^{-1} \hbox {d}G. \end{aligned}$$

Computing

2.36 $$\begin{aligned} G^{-1} \hbox {d}G= \frac{i\ell \hbox {d}\mathbf {x} \cdot \varvec{\tau } + i \mathbf {x}\times \hbox {d}\mathbf {x}\cdot \varvec{\tau }}{\ell ^2 +r^2}, \end{aligned}$$

we have

2.37 $$\begin{aligned} \hbox {d}G G^{-1} + G^{-1} \hbox {d}G= \frac{2i\ell \hbox {d}\mathbf {x} \cdot \varvec{\tau }}{\ell ^2 +r^2}, \quad \hbox {d}G G^{-1} - G^{-1} \hbox {d}G = -\frac{2 i \mathbf {x}\times \hbox {d}\mathbf {x}\cdot \varvec{\tau }}{\ell ^2 +r^2}. \end{aligned}$$

With $$ 2 \hbox {d}\varOmega = \mathbf {x}\cdot \hbox {d}\mathbf {x}/\ell $$ and

2.38 $$\begin{aligned} \star (\mathbf {x}\cdot \hbox {d}\mathbf {x} \wedge \hbox {d}\mathbf {x}\cdot \varvec{\tau })= \hbox {d}\mathbf {x}\times \mathbf {x} \cdot \mathbf { \tau }, \end{aligned}$$

one deduces (2.35) and hence (2.34). $\square$

### Magnetic Dirac operators and their zero-modes

In the frame (2.19) for a 3-sphere of radius $\ell$, a global gauge potential for the spin connection is

2.39 $$\begin{aligned} \Gamma _{S^3} = \frac{1}{2} h^{-1}\hbox {d}h, \end{aligned}$$

Thus, the Dirac operator associated with the frame (2.19) is2.40Minimal coupling to an abelian gauge potential $A =A_1 \sigma _1 + A_2 \sigma _2 + A_3\sigma _3$ gives2.41The Dirac operator on $\mathbb {R}^3$ associated with the frame (2.22) and minimally coupled to an abelian gauge potential $A= A_1\hbox {d}x_1 + A_2 \hbox {d}x _2 + A_3\hbox {d}x_3$ is2.42where we used $\partial _1= \partial /\partial x_1$ etc.

As we saw in the previous section, the frame (2.19) pulled back to $\mathbb {R}^3$ via *H* and the flat frame (2.22) are related by a rotation with *G*, a reflection and rescaling by (2.29). This implies a simple relation between the zero-modes of the Dirac operators on $S^3$ and $\mathbb {R}^3$.

#### Lemma 2.2

If $\varPsi : S^3\rightarrow \mathbb {C}^2$ is a zero-mode of the Dirac operator (2.41) on $S^3$ coupled to the *U*(1) gauge field *A*, then

2.43 $$\begin{aligned} \varPsi _H = G\varOmega ^{-1} H^*\varPsi \end{aligned}$$

is a zero-mode of the Dirac operator  on Euclidean 3-space coupled to the connection $H^*A$.

#### Proof

Since the spin connection in the frame (2.22) is manifestly zero, it follows from the equivariance of the Dirac operator under scaling and frame rotations that pulling back zero-modes of the Dirac operator on $S^3$ to $\mathbb {R}^3$ and applying the transformation $G\varOmega ^{-1}$ gives zero-modes of the Dirac operator on $\mathbb {R}^3$ in the flat frame (2.22). It is instructive to check this explicitly. The pull-back of the spin connection is

2.44 $$\begin{aligned} H^*\Gamma _{S^3} = \frac{1}{2} H^{-1}\hbox {d}H. \end{aligned}$$

Thus, using (2.33),

2.45 $$\begin{aligned} d + \frac{1}{2} H^{-1} \hbox {d}H =\varOmega G^{-1}\left( d+ \frac{1}{2}\left( G \hbox {d}G^{-1} + G^{-1} \hbox {d}G\right) + \varOmega ^{-1} \hbox {d}\varOmega \right) \varOmega ^{-1} G. \end{aligned}$$

Combining (2.37) and

2.46 $$\begin{aligned} \varOmega ^{-1}\hbox {d}\varOmega =\frac{2\mathbf {x}\cdot \hbox {d}\mathbf {x}}{\ell ^{2}+r^{2}}, \end{aligned}$$

one checks that

2.47 $$\begin{aligned} \tau ^{j}\iota _{\partial _j}\left( \frac{1}{2}\left( G \hbox {d}G^{-1} + G^{-1} \hbox {d}G\right) + \varOmega ^{-1} \hbox {d}\varOmega \right) =-\frac{2\mathbf {x}\cdot \varvec{\tau }}{\ell ^{2}+r^{2}}+\frac{2\mathbf {x}\cdot \varvec{\tau }}{\ell ^{2}+r^{2}}=0. \end{aligned}$$

Combining these results, one checks that the pull-back of the Dirac operator on $S^3$ coupled to the spin connection and the abelian connection *A* in the frame (2.22) is2.48which implies the claimed relation between zero-modes of  and . Note that the components of the pull-back abelian connection relative to the frame (2.22) are related to the components in the expansion $A=\mathbf {A}\cdot \varvec{\sigma }$ via

2.49 $$\begin{aligned} H^*A = \mathbf {A}_H\cdot \hbox {d}\mathbf {x}, \quad \mathbf {A}_H\cdot \varvec{\tau } =-\frac{1}{\varOmega } G^{-1} H^{*}\mathbf {A}\cdot \varvec{\tau }G. \end{aligned}$$

$$\square $$

This lemma can be used to construct magnetic zero-modes on $\mathbb {R}^3$ from magnetic zero-modes on $S^3$. For the family constructed explicitly by Loss and Yau in [1], which we call linear in this paper, this was observed in [6] and elaborated in [7], where this family was obtained from eigenmodes of the Dirac operator on $S^3$. The corresponding argument for the family of vortex zero-modes is one of the main results of our Sect. 3.2.

We review the construction of the linear zero-modes very briefly here because we will need them later in this paper, expressed in our conventions for parameterising $S^3$ in terms of two complex variables. We define the functions

2.50 $$\begin{aligned} Y^j_{sm}(z_1,z_2)= & {} C_{jms} \sum _k\frac{(-1)^{-k} }{(j+m-k)!k!(j-s -k)!(s -m + k)!}\nonumber \\&\times \, z_1^{s-m+k}z_2^{j+m-k}\bar{z}_1^k\bar{z}_2^{j-s-k}, \end{aligned}$$

where $C_{jms}$ is an overall normalisation constant

2.51 $$\begin{aligned} C_{jms}=(-1)^{j-s} \left( (j+s)!(j-s)!(j+m)!(j-m)!\right) ^{\frac{1}{2} }, \end{aligned}$$

and

2.52 $$\begin{aligned} j\in \frac{1}{2} \mathbb {N}^0, \quad s, m=-j,-j+1,\ldots ,j-1,j. \end{aligned}$$

The summation index *k* runs over the values so that the factorials are well defined. These functions are orthonormal and satisfy

2.53 $$\begin{aligned} \varDelta _{S^3}Y^j_{sm} = -j(j+1)Y^j_{sm}, \ \ iZ_3Y^j_{sm} = mY^j_{sm}, \ \ iX_3Y^j_{sm} = sY^j_{sm}. \end{aligned}$$

as well as

2.54 $$\begin{aligned} iX_+ Y^j_{sm}{=} \sqrt{j(j{+}1)-s(s{+}1)}Y^j_{s+1,m}, \quad iX_- Y^j_{sm}= \sqrt{j(j{+}1)-s(s{-}1)}Y^j_{s-1,m}. \end{aligned}$$

Using the explicit expression for the Dirac operator given in (2.40), one deduces that

2.55 $$\begin{aligned} \varPsi ^{j+}_{sm} = \frac{1}{2j+1}\begin{pmatrix} \sqrt{j+s+1} Y^j_{sm}\\ \sqrt{j-s} Y^j_{s+1,m}\end{pmatrix} \end{aligned}$$

is an eigenspinor of  with eigenvalue

2.56 $$\begin{aligned} \lambda _+= \frac{1}{\ell }\left( \frac{1}{2} +\left( 2j+1 \right) \right) , \end{aligned}$$

and that

2.57 $$\begin{aligned} \varPsi ^{j-}_{sm} = \frac{1}{2j+1}\begin{pmatrix} -\sqrt{j-s} Y^j_{sm}\\ \sqrt{j+s+1} Y^j_{s+1,m}\end{pmatrix} \end{aligned}$$

is an eigenspinor of  with eigenvalue

2.58 $$\begin{aligned} \lambda _-= \frac{1}{\ell }\left( \frac{1}{2} -\left( 2j+1 \right) \right) . \end{aligned}$$

The degeneracy of each eigenvalue is $(2j+1)(2j+2)$.

We can now use a trick introduced by Loss and Yau [1] to obtain zero-modes of the gauged Dirac operator from general eigenmodes of the ungauged Dirac operator. Setting

2.59 $$\begin{aligned} \frac{2}{\ell } A_i = \lambda \frac{\varPsi ^\dagger \tau _i \varPsi }{\varPsi ^\dagger \varPsi }, \quad i=1,2,3, \end{aligned}$$

where $\varPsi \ne 0$ and using $\varPsi ^\dagger \tau _i \varPsi \tau _i =2 \varPsi \varPsi ^\dagger -{\varPsi ^\dagger \varPsi }\mathbb {I}$, one then has

2.60 $$\begin{aligned} \frac{2}{\ell }\mathbf {A}\cdot \varvec{\tau }\varPsi =\lambda \varPsi . \end{aligned}$$

With $A= A_i\sigma _i$, this implies2.61In general, one needs to check the validity of this result at the zeros of $\varPsi$. We will do this in our application of linear zero-modes later. Assuming the zeros are dealt with, we can then apply Lemma 2.2 to obtain magnetic zero-modes on Euclidean 3-space of the form

2.62 $$\begin{aligned} \varPsi _H= G\varOmega ^{-1} H^* \varPsi ^{j\pm }_{sm}. \end{aligned}$$

## Vortex equations and magnetic zero-modes

### Vortex equations on $S^3$

We are now ready to introduce the 3-dimensional geometries which will lead us to the smooth vortex zero-modes promised in the Introduction and provide the link with vortices on the 2-sphere. First, we define vortex configurations on the 3-sphere.

#### Definition 3.1

Let *n* be a positive integer, *A* be a 1-form on $S^3$, and $\varPhi : S^3\rightarrow \mathbb {C}$ be a complex-valued function. We say that the pair $(\varPhi ,A)$ is a vortex configuration on $S^3$ with vortex number $2n-2$ if the following conditions hold:

1. Normalisation :
  3.1 $$\begin{aligned} A(X_3)=n-1, \end{aligned}$$
2. Equivariance:
  3.2 $$\begin{aligned} {{\mathcal {L}}}_{X_3}A =0, \quad i{{\mathcal {L}}}_{X_3}\varPhi =(n-1)\varPhi , \end{aligned}$$
3. Vortex equations:
  3.3 $$\begin{aligned} (\hbox {d}\varPhi +i A\varPhi )\wedge \sigma = 0, \quad F_A = \frac{i}{2} (|\varPhi |^2-1)\bar{\sigma } \wedge \sigma , \end{aligned}$$
  where $$F_A=\hbox {d}A$$.

The definition is such that vortex configurations are mapped into vortex configurations by abelian gauge transformations of the form

3.4 $$\begin{aligned} (\varPhi ,A) \mapsto (e^{-i\alpha }\varPhi ,A+\hbox {d}\alpha ), \quad \alpha \in C^\infty (S^3), \quad X_3\alpha =0. \end{aligned}$$

Our vortex configurations on $$S^3$$ can be interpreted as an equivariant description of vortices on $$S^2$$ as follows. The normalisation condition (3.1) means that *iA* may be viewed as the connection 1-form on the total space $$S^3/\mathbb {Z}_{2n-2}$$ (Lens space) of a *U*(1) bundle over $$S^2$$ of degree $$2n-2$$. Comparing with (2.17) and referring to [11] for details, the equivariance requirement (3.2) means that $$\varPhi $$ is the equivariant form of a section of the associated line bundle (the ($$2n-2)$$th power of the hyperplane bundle). In fact, we will show in Sect. 4.1, Lemma 4.1, that the vortices on $$S^2$$ which are equivariantly described by our vortex configurations are Popov vortices.

We note that contracting the first vortex equation with $(X_3,X_-)$ and using (2.14) gives $X_3\varPhi +iA(X_3)\varPhi =0$, which is satisfied by virtue of the normalisation and equivariance condition. Contracting it with $(X_+,X_-)$ gives

3.5 $$\begin{aligned} X_+\varPhi +iA(X_+)\varPhi =0. \end{aligned}$$

We will return to this equation later in this section and also in Sect. 4.1. However, we first show that the vortex equations on $S^3$ can be interpreted in terms of a flat non-abelian gauge field.

The following theorem shows that any vortex configuration can be expressed in terms of the pull-back of the Maurer–Cartan form $h^{-1}\hbox {d}h$ on *SU*(2) via a bundle map $U:S^3 \rightarrow S^3$ of the Hopf fibration covering a rational map $S^2\rightarrow S^2$. Since the Maurer–Cartan form encodes the frame (2.19) of the round 3-sphere, its pull-back encodes the pull-back of the round metric with *U*. In that sense, this result is a 3-dimensional version of Baptista’s interpretation of vortices as deformed 2-dimensional geometry.

#### Theorem 3.2

A vortex configuration of degree $2n-2$ on $S^3$ determines a gauge potential for a flat *su*(2) connection on $S^3$ satisfying the normalisation condition

3.6 $$\begin{aligned} \mathcal {A}(X_3)=nt_3 \end{aligned}$$

via the following expression:

3.7 $$\begin{aligned} \mathcal {A}=(A+\sigma _3)t_3 +\frac{1}{2}( \varPhi \sigma t_-+ \bar{\varPhi }\bar{\sigma } t_+). \end{aligned}$$

A gauge potential for a flat *su*(2) connection on $S^3$ satisfying (3.6) and of the form (3.7) can be trivialised as $\mathcal {A}=U^{-1}\hbox {d}U$, where $U:S^3\rightarrow S^3$ has degree $n^2$ and is a bundle map of the Hopf fibration, covering a rational map $R:S^2\rightarrow S^2$ of degree *n*. Up to a *U*(1) gauge transformation (3.4), one can choose the bundle map *U* to have the form

3.8 $$\begin{aligned} U:(z_{1},z_{2})\mapsto \frac{1}{\sqrt{|P_{1}|^{2}+|P_{2}|^{2}}} \begin{pmatrix} P_{1} &{} \quad -\bar{P}_2 \\ P_{2} &{}\quad \bar{P}_1\end{pmatrix}, \end{aligned}$$

where $P_1,P_2$ are homogeneous polynomials of degree *n* with no common zeros

3.9 $$\begin{aligned} P_1= a_0z_1^n + a_1z_1^{n-1}z_2+\cdots + a_n z_2^n, \quad P_2= b_0z_1^n + b_1z_1^{n-1}z_2+\cdots + b_n z_2^n, \end{aligned}$$

and $a_0,b_0,a_n,b_n$ all nonzero.

The vortex configuration $(\varPhi ,A)$ can be computed from the bundle map *U* via

3.10 $$\begin{aligned} U^*\sigma =\varPhi \sigma , \quad A= U^*\sigma _3-\sigma _3, \end{aligned}$$

and is given in terms of $P_1,P_2$ by

3.11 $$\begin{aligned} \varPhi = \frac{P_1\partial _2 P_2 - P_2 \partial _2 P_1}{z_1(|P_1|^2 + |P_2|^2)}, \end{aligned}$$

and

3.12 $$\begin{aligned} A= (n-1)\sigma _3 + \frac{i}{2} X_-\ln \left( |P_1|^2 + |P_2|^2\right) \sigma - \frac{i}{2} X_+\ln \left( |P_1|^2 + |P_2|^2\right) \bar{\sigma }. \end{aligned}$$

Our condition on $a_0,b_0,a_n,b_n$ will turn out to be convenient in the discussion of Popov vortices in Sect. 4.2 and facilitates comparison with the treatment in [9].

#### Proof

Suppose $(\varPhi ,A)$ is a vortex configuration of degree $2n-2$. It is easy to check that, for a gauge potential of the form (3.7), the normalisation (3.1) implies (3.6). The flatness condition $\hbox {d}\mathcal {A}+ \mathcal {A}\wedge \mathcal {A}=0$ for a gauge potential of the form (3.7) is equivalent to

3.13 $$\begin{aligned} \hbox {d}(\varPhi \sigma ) + i(A+\sigma _3) \wedge \varPhi \sigma =0, \quad dA = \frac{i}{2}(|\varPhi |^2 -1)\bar{\sigma }\wedge \sigma , \end{aligned}$$

which, using (2.7), is equivalent to the vortex equations (3.3). The equivariance condition (3.2) for vortex configurations is equivalent to

3.14 $$\begin{aligned} {{\mathcal {L}}}_{X_3} \mathcal {A}= n[\mathcal {A},t_3], \end{aligned}$$

but this holds automatically for a flat gauge potential satisfying the normalisation (3.6) since, for a flat gauge field,

3.15 $$\begin{aligned} {{\mathcal {L}}}_{X_3} \mathcal {A}= D_\mathcal {A}\mathcal {A}(X_3). \end{aligned}$$

A flat and smooth *SU*(2) gauge potential $\mathcal {A}$ on $S^3$ can always be globally trivialised in terms of a function $U:S^3 \rightarrow SU(2)$ as $\mathcal {A}=U^{-1}\hbox {d}U$. We now show that the vortex form (3.7) and the normalisation (3.6) force the trivialising map to be a bundle map covering a rational map of degree *n*. The normalisation (3.6) requires

3.16 $$\begin{aligned} X_3 U =nUt_3, \quad \text {or} \quad U(he^{\gamma t_3}) = U(h)e^{n\gamma t_3}, \quad \gamma \in [0,4\pi ). \end{aligned}$$

This equivariance condition has important topological consequences. It implies that the map $\pi \circ U$ is constant along fibres of the Hopf fibration and determines a map $S^2 \rightarrow S^2$; in the parameterisation of *U* in terms of two functions $P_1,P_2$ which do not vanish simultaneously as in (3.8) (but without assuming that $P_1,P_2$ are polynomials) this map is simply the quotient $P_2/P_1$. In terms of our stereographic coordinate *z* for $S^2$ and the section *s* in (2.16) we define

3.17 $$\begin{aligned} R = \pi \circ U \circ s \end{aligned}$$

and have the following commutative diagram (where we have not carefully distinguished between $S^2$ and our coordinate chart $\mathbb {C}$ for it):3.18By virtue of (3.16), the map $\pi \circ U:S^3\rightarrow S^2$ has Hopf number $n^2$: the pre-image of any point on $S^2$ is an *n*-fold cover of a circle which links with each of the *n* circles in the pre-image of another point exactly once. It follows that the map *U* has degree $n^2$ and the map *R* covered by *U* has degree *n*.

Continuing in a parameterisation of *U* in terms of two functions $P_1,P_2$ but still not assuming that $P_1,P_2$ are polynomials, the condition (3.16) implies

3.19 $$\begin{aligned} 2i X_3P_1 = nP_1, \quad 2i X_3P_2 = nP_2. \end{aligned}$$

Since

3.20 $$\begin{aligned} U^{-1} \hbox {d}U = U^*\sigma _3 t_3 +\frac{1}{2}( U^*\sigma t_-+ U^*\bar{\sigma } t_+), \end{aligned}$$

we obtain a potential in the vortex gauge (3.7) if and only if

3.21 $$\begin{aligned} U^*\sigma = \varPhi \sigma \end{aligned}$$

for some function $\varPhi :S^3 \rightarrow \mathbb {C}$. Using (2.14), we therefore need to show that

3.22 $$\begin{aligned} (U^{*}\sigma )(X_{3})=0, \quad ( U^{*}\sigma )(X_{+}) =0. \end{aligned}$$

The first of these follows from (3.19), since

3.23 $$\begin{aligned} (U^{*}\sigma )(X_{3})= \frac{2i}{|P_1|^2+ |P_2|^2}(P_1X_3P_2-P_2X_3P_1). \end{aligned}$$

To analyse the second condition, note that

3.24 $$\begin{aligned} (U^{*}\sigma )(X_{+})=\frac{2i}{|P_1|^2+ |P_2|^2}(P_{1}X_{+}P_{2}-P_{2}X_{+}P_{1})=\frac{2i}{|P_1|^2+ |P_2|^2}P_{1}^{2}X_{+}\left( \frac{P_{2}}{P_{1}}\right) , \end{aligned}$$

with the last equality holding where $P_1\ne 0$. As noted above, the ratio $P_2/P_1$ defines a function (section of the trivial bundle $H^0$) on $S^2$. According to the commutative diagram (2.18), $X_+(P_2/P_1)=0$ means that the pull-back $R=s^*(P_2/P_1)$ is, in fact, a holomorphic function where it is defined. Thus, *R* has to be a holomorphic map $S^2\rightarrow S^2$ of degree *n*, which means it must be a rational map, as claimed.

These conditions are clearly satisfied when $P_1$ and $P_2$ are the homogeneous polynomials given in (3.8). In that case, the rational map is explicitly given by

3.25 $$\begin{aligned} R(z)=\frac{p_2(z)}{p_1(z)}, \end{aligned}$$

where

3.26 $$\begin{aligned} p_1(z)= a_0+a_1z+\cdots +a_nz^n, \quad p_2(z)= b_0+b_1z+\cdots +b_nz^n. \end{aligned}$$

In order for (3.25) to be a map of degree *n* we require at least one of $a_n,b_n$ to be nonzero (so that the maximum of the degrees of $p_1$ and $p_2$ is *n*) and at least one of $a_0, b_0$ to be nonzero (so that we cannot reduce the degree by cancellation). We can then arrange for all of $a_0, b_0,a_n,b_n$ to be nonzero by left-multiplying *U* with a constant *SU*(2) matrix if necessary; this does not affect $\mathcal {A}$ and therefore leaves the vortex configuration unchanged.

Fixing *U* to be the trivialisation in terms of the polynomials $P_1,P_2$ in (3.8), we can define a new trivialisation

3.27 $$\begin{aligned} {\tilde{U}} = U e^{\alpha t_3}, \quad \alpha \in C^\infty (S^3),\quad X_3 \alpha =0. \end{aligned}$$

This also satisfies (3.16) and leads to the same rational map *R*. The non-abelian gauge potential ${\tilde{\mathcal {A}}}= {\tilde{U}}^{-1} d{\tilde{U}}$ differs from $\mathcal {A}= U^{-1} \hbox {d}U$ by the gauge transformation (3.4), as claimed.

Continuing with $P_1$ and $P_2$ being homogeneous polynomials in $z_1,z_2$ of degree *n*, we obtain the claimed formula for the vortex field $\varPhi$ from

3.28 $$\begin{aligned} \varPhi = (U^{*}\sigma )(X_{-}) = \frac{P_1\partial _2 P_2 - P_2 \partial _2 P_1}{z_1\left( |P_1|^2 + |P_2|^2\right) }, \end{aligned}$$

noting that

3.29 $$\begin{aligned} \frac{P_1\partial _2 P_2 - P_2 \partial _2 P_1}{z_1} \end{aligned}$$

is a homogeneous polynomial in $z_1,z_2$ of degree $2n-2$ and non-singular: the term of order $z_2^{2n-1}$, which could potentially cause a singularity when divided by $z_1$, vanishes.

The derivation of the expression for *A* is a straightforward calculation, which makes use of

3.30 $$\begin{aligned} (z_1\partial _1 + z_2\partial _2) \left( |P_1|^2+|P_2|^2\right) = n\left( |P_1|^2+|P_2|^2\right) . \end{aligned}$$

One finds

3.31 $$\begin{aligned} U^*\sigma _3(X_3)= & {} n, \;\; U^*\sigma _3(X_+)=-iX_{+}\ln \left( |P_1|^2 +|P_2|^2\right) , \nonumber \\ U^*\sigma _3(X_-)= & {} iX_{-}\ln \left( |P_1|^2+|P_2|^2\right) , \end{aligned}$$

which, with (2.14), implies (3.12). $\square$

In order to make contact with discussions in the literature related to the potential *A* we note an expression for *A* in terms of polar coordinates, for later use.

#### Lemma 3.3

Suppose $(\varPhi ,A)$ is a vortex configuration of vortex number $2n-2$ on $S^3$ and consider the modulus-argument parameterisation

3.32 $$\begin{aligned} \varPhi = e^{\frac{M}{2}+i\chi }, \end{aligned}$$

valid away from the (generically $2n-2$) zeros of $\varPhi$. Then, the gauge potential *A* in (3.10) can be expressed via the formula

3.33 $$\begin{aligned} A= -\frac{\ell }{4} \star (\sigma _3 \wedge \hbox {d}M) -\hbox {d}\chi , \end{aligned}$$

valid away from the zeros of $\varPhi$.

#### Proof

Observe that, away from the zeros of $\varPhi$, we can write (3.12) as

3.34 $$\begin{aligned} A= (n-1)\sigma _3 - \frac{i}{2}X_-\ln \bar{\varPhi }\sigma + \frac{i}{2}X_+\ln \varPhi \bar{\sigma }. \end{aligned}$$

Inserting the parameterisation (3.32) leads to

3.35 $$\begin{aligned} A -(n-1)\sigma _3= \left( - \frac{i}{4} X_-M - \frac{1}{2} X_-\chi \right) \sigma + \left( \frac{i}{4} X_+ M -\frac{1}{2} X_+\chi \right) \bar{\sigma }. \end{aligned}$$

With the Hodge-$\star$ relative to the orientation (2.21), we have

3.36 $$\begin{aligned} \star (\sigma _3 \wedge \sigma ) = i\frac{2}{\ell } \sigma , \quad \star (\sigma _3 \wedge \bar{\sigma }) = -i\frac{2}{\ell } \bar{\sigma }, \end{aligned}$$

so that

3.37 $$\begin{aligned} - \frac{i}{4} X_-M \sigma + \frac{i}{4} X_+ M\bar{\sigma }=-\frac{\ell }{4} \star (\sigma _3 \wedge dM), \end{aligned}$$

where we have used that for any differentiable $f:S^3\rightarrow \mathbb {C}$,

3.38 $$\begin{aligned} df =\frac{1}{2} X_-f \sigma + \frac{1}{2} X_+f \bar{\sigma } + X_{3}f\sigma _{3}, \end{aligned}$$

Turning to the terms involving $\chi$, using (3.38) and deducing from (3.2) that $X_3\chi = 1-n$, we conclude that

3.39 $$\begin{aligned} \hbox {d}\chi =\frac{1}{2} X_-\chi \sigma + \frac{1}{2} X_+ \chi \bar{\sigma } - (n-1)\sigma _3. \end{aligned}$$

Combining (3.35) with (3.37) and (3.39) we arrive at the claimed expression for the gauge potential (3.10) in terms of the modulus and argument of the field $\varPhi$. $\square$

### Magnetic zero-modes from vortices

We are now ready to explain how one can construct magnetic zero-modes of the Dirac operator on the 3-sphere and on Euclidean 3-space from vortex configurations on the 3-sphere. We define spinorial vortex zero-modes as follows.

#### Definition 3.4

A pair $(\varPsi ,A)$ of a spinor $\varPsi$ and a 1-form *A* on $S^3$ is said to be a vortex zero-mode of the Dirac equation on $S^3$ if3.40where $\star$ is the Hodge star operator on $S^3$ with respect to the metric (2.20) and orientation (2.21).

#### Theorem 3.5

Suppose $(\varPhi ,A)$ is a vortex configuration on $S^3$. Then, the pair

3.41 $$\begin{aligned} \varPsi = \begin{pmatrix} \varPhi \\ 0 \end{pmatrix}, \quad A'= A+\frac{3}{4} \sigma _3, \end{aligned}$$

is a vortex zero-mode $(\varPsi ,A')$ on $S^3$.

#### Proof

The spinor given in the theorem is a zero-mode of the gauged Dirac equation if

3.42 $$\begin{aligned} \left( iX_3-A'_3 +\frac{3}{4} \right) \varPhi = 0 \quad \text {and} \quad X_+\varPhi + iA'_+\varPhi =0. \end{aligned}$$

However, $A'_3= A'(X_3)= (n-1)+ \frac{3}{4}$ so that the first of these equations follows from (3.2). The second follows from $A'(X_+) = A(X_+)$ and (3.5). Turning to the nonlinear equation, we note that, for a spinor of the form given in the theorem,

3.43 $$\begin{aligned} \frac{4i}{\ell }\star \varPsi ^\dagger h^{-1}\hbox {d}h\varPsi = \frac{4i}{\ell }|\varPhi |^2 \star \left( -\frac{i}{2}\sigma _3\right) = |\varPhi |^2\sigma _2\wedge \sigma _1. \end{aligned}$$

Moreover,

3.44 $$\begin{aligned} F_{A'} = F_A + \frac{3}{4} \sigma _2\wedge \sigma _1 = \left( |\varPhi |^2 - \frac{1}{4} \right) \sigma _2\wedge \sigma _1, \end{aligned}$$

so that the nonlinear equation in the definition of a vortex zero-mode follows.$\square$

We can pull back the vortex zero-modes of the Dirac equation on $S^3$ to $\mathbb {R}^3$ using Lemma 2.2, but we also need to understand how the nonlinear equation behaves under this pull-back. It turns out that the resulting equations take their simplest form in vector notation for gauge potentials and their magnetic fields, i.e. when expanding a 1-form on $\mathbb {R}^3$ as $A=\mathbf {A}\cdot \hbox {d}\mathbf {x}$ and defining the magnetic field vector field via $dA=\frac{1}{2} \epsilon _{jkl} B_j \hbox {d}x_k \wedge \hbox {d}x_l$ or $\mathbf {B}=\nabla \times \mathbf {A}$.

The magnetic field corresponding to the inhomogeneous term is given by

**Fig. 2:**
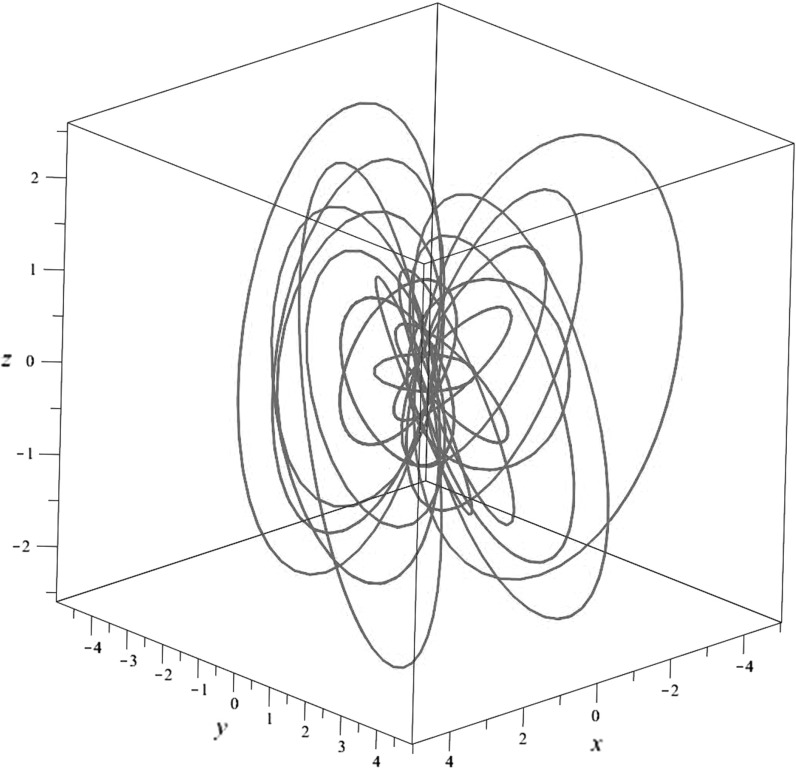
A plot of some of the integral curves of the background field $\mathbf {b}$ given in (3.45)

3.45 $$\begin{aligned} \frac{1}{4}H^*(\sigma _1\wedge \sigma _2)= & {} \frac{4\ell ^2}{(\ell ^2+r^2)^2} \star _{\mathbb {R}^3}\theta _3= \frac{1}{2} \epsilon _{jkl} b_{j} \hbox {d}x_k\wedge \hbox {d}x_l, \nonumber \\ \mathbf {b}= & {} \frac{4\ell ^{2}}{(\ell ^{2}+r^{2})^{3}}\begin{pmatrix} 2(x_1x_3-\ell x_2)\\ 2(x_2x_3+\ell x_1)\\ \ell ^{2}-r^{2}+2x_3^{2} \end{pmatrix}, \end{aligned}$$

where we used (2.32). The integral lines of $\mathbf {b}$ are the fibres of the Hopf fibration (2.15); they are plotted in Fig. 2.

We claim that vortex zero-modes of the Dirac equation pull back to solutions of the following coupled equations in $\mathbb {R}^3$:3.46We state this result as follows.

#### Corollary 3.6

Any pair of homogeneous polynomials $P_1,P_2:\mathbb {C}^2 \rightarrow \mathbb {C}$ of the same degree and without common zeros uniquely determines a smooth and square-integrable magnetic zero-mode of the Dirac operator in Euclidean 3-space which satisfies the coupled equations (3.46).

#### Proof

Combining $P_1$ and $P_2$ into an *SU*(2) matrix yields a vortex configuration $(\varPhi ,A)$ on $S^3$ according to the prescription of Theorem 3.2. Such a vortex configuration defines a vortex zero-mode $\varPsi$ of the Dirac operator on $S^3$ coupled to $A'= A+ \frac{3}{4} \sigma _3$ according to Theorem 3.5. Implementing the conformal change to $\mathbb {R}^3$ according to Lemma 2.2 produces the magnetic zero-mode

3.47 $$\begin{aligned} \varPsi _H= \varOmega ^{-1} G H^*\varPsi = \varOmega ^{-1} G\begin{pmatrix}H^*\varPhi \\ 0 \end{pmatrix} \end{aligned}$$

on $\mathbb {R}^3$ of the Dirac operator coupled to $H^*A'=\mathbf {A'}_H\cdot \hbox {d}\mathbf {x}$.

We also need to understand the pull-back of the nonlinear equation in (3.40). The quadratic term in the zero-mode $\varPsi$ on $S^3$ is

3.48 $$\begin{aligned} \frac{4i}{\ell }\star \varPsi ^\dagger h^{-1}\hbox {d}h\varPsi = \frac{1}{2} \epsilon _{jkl} \varPsi ^\dagger \tau _j \varPsi \sigma _l\wedge \sigma _k, \end{aligned}$$

and using (2.30) and (2.31) we deduce that pulling back with *H* yields

3.49 $$\begin{aligned} H^*\left( \frac{4i}{\ell }\star \varPsi ^\dagger h^{-1}\hbox {d}h\varPsi \right)= & {} \frac{1}{2\varOmega ^2} \epsilon _{jkl} H^*\varPsi ^\dagger G^{-1}\tau _j G H^*\varPsi \hbox {d}x_l\wedge \hbox {d}x_k \nonumber \\= & {} -\frac{1}{2} \varPsi ^\dagger _H\tau _j\varPsi _H\epsilon _{jkl} \hbox {d}x_k\wedge \hbox {d}x_l, \end{aligned}$$

where $\varPsi _H$ is related to $\varPsi$ as defined in (2.43). Finally, expanding the pull-back of the field strength in the same coordinates

3.50 $$\begin{aligned} H^*F_{A'}=\frac{1}{2} \epsilon _{jkl} (B'_H)_j \hbox {d}x_k \wedge \hbox {d}x_l, \quad \text {so that} \quad \mathbf {B'}_H =\nabla \times \mathbf {A'}_H, \end{aligned}$$

the nonlinear equation in the definition 3.4 pulls back to

3.51 $$\begin{aligned} \mathbf {B'}_H = - \varPsi _H^\dagger \varvec{\tau } \varPsi _H + \mathbf {b}, \end{aligned}$$

as claimed.

The spinor $\varPsi _H$ in (3.47) and the gauge potential $H^*A'$ are manifestly smooth, being the pull-back with smooth maps of smooth functions on $S^3$. As the pull-back of a smooth function on $S^3$, $H^*\varPhi$ is bounded and has a (finite) limit as $r\rightarrow \infty$. It follows that

3.52 $$\begin{aligned} |\varPsi _H^\dagger \varPsi _H| \le \frac{C}{\varOmega ^2}, \end{aligned}$$

for some positive constant *C*, which ensures that $\varPsi _H$ is square-integrable with respect to the Euclidean measure (2.23). Since $|\varPsi ^\dagger \varvec{\tau } \varPsi |= |\varPsi ^\dagger \varPsi |$ for any spinor $\varPsi$, it follows that the vector field $\varPsi _H^\dagger \varvec{\tau } \varPsi _H$ is also square-integrable. The square-integrability of $\mathbf {B'}_H$ then follows from the square-integrability of $\mathbf {b}$ and the relation (3.51). $\square$

The coupled equations (3.46) have appeared in the literature in various contexts and deserve a few comments. There are various ways of stating these equations. Rescaling the spinor by a factor of $\sqrt{2}$ leaves the linear equations unchanged, but changes the quadratic term in the nonlinear equation into the spin density

3.53 $$\begin{aligned} \varvec{\Sigma } =\frac{1}{2} \varPsi ^\dagger \varvec{\tau } \varPsi . \end{aligned}$$

Changing to the charge conjugate spinor

3.54 $$\begin{aligned} \varPsi ^c = i\tau _2\varPsi , \end{aligned}$$

turns our equations into the equivalent set of equations3.55The equations (3.46) have been discussed in the literature as the dimensionally reduced Freund equations [2], while their charge conjugates have appeared as the variational equations of a particular Dirac–Chern–Simons action [4].

The magnetic field on $\mathbb {R}^3$ obtained from the pair of complex polynomials $P_1,P_2$ can be written in terms of the Hopf map $\pi$ and the maps *H* (2.26) and *U* (3.8) as

3.56 $$\begin{aligned} F=(\pi \circ U \circ H)^*\mathcal {R}, \end{aligned}$$

where $\mathcal {R}$ is the area form on the 2-sphere of unit radius (4.7), which will play an important role in the next section. This is an example of the magnetic field introduced by Rañada in [13] and discussed more recently in [14]. It has interesting topological properties inherited from those of the map $U:S^3\rightarrow S^3$, which, as explained after diagram 3.18, has topological degree $n^2$. As also explained there, $\pi \circ U:S^3\rightarrow S^2$ has Hopf number $n^2$. As discussed in [15], this implies that the magnetic field (3.56) has linking number one and (magnetic) helicity $n^2$.

In order to compare with solutions for (3.46) previously obtained in the literature, we also pull back the modulus-argument expression (3.33) to $\mathbb {R}^3$ to find

3.57 $$\begin{aligned} H^{*}A=-\frac{\ell }{4}H^{*}\left( \star (\sigma _{3}\wedge \hbox {d}M)\right) -\hbox {d}(H^*\chi ). \end{aligned}$$

The operation

3.58 $$\begin{aligned} \star \sigma _3\wedge \, : \Lambda ^1(S^3)\rightarrow \Lambda ^1(S^3) \end{aligned}$$

is linear; it annihilates $\sigma _3$ and acts as a complex structure on the cotangent space orthogonal to $\sigma _3$ by mapping

3.59 $$\begin{aligned} \star \sigma _3\wedge \, : \sigma \mapsto (2 i/ \ell ) \sigma . \end{aligned}$$

It pulls back to the map $(\theta _1+i\theta _2) \mapsto (2 i/ \ell )( \theta _1+i\theta _2)$, which we can write as

3.60 $$\begin{aligned} -\frac{2}{\ell }\star _{\mathbb {R}^3}\theta _3\wedge \,: \Lambda ^1(\mathbb {R}^3)\rightarrow \Lambda ^1(\mathbb {R}^3). \end{aligned}$$

Therefore,

3.61 $$\begin{aligned} H^*A=-\frac{1}{2}\star _{\mathbb {R}^{3}}\left( \hbox {d} (H^*M) \wedge \theta _{3}\right) -\hbox {d} (H^*\chi ). \end{aligned}$$

In [1], Loss and Yau showed that, for spinors on $\mathbb {R}^3$ whose spin density (3.53) has vanishing divergence, one can always find a gauge field *A* so that the given spinor is a zero-mode of the Dirac operator on $\mathbb {R}^3$ coupled to *A*. They gave an explicit formula, valid where the spinor does not vanish:

3.62 $$\begin{aligned} A_\varPsi = -\frac{1}{2} \star \frac{\hbox {d} (\varPsi ^\dagger \hbox {d} \mathbf {x} \cdot \varvec{\tau } \varPsi )}{\varPsi ^\dagger \varPsi } - \frac{ \text {Im}(\varPsi ^\dagger d \varPsi )}{\varPsi ^\dagger \varPsi }. \end{aligned}$$

The relation to our expression (3.61) is as follows.

#### Lemma 3.7

Let $(\varPhi ,A)$ be a vortex configuration on $S^3$, and $\varPsi _H$ the corresponding zero-mode of the Dirac operator on $\mathbb {R}^3$ given in (3.47). Then, the spin density of $\varPsi _H$ is divergenceless, and the corresponding Loss–Yau potential is given by

3.63 $$\begin{aligned} A_{\varPsi _H} = H^*A+ \frac{3}{4} H^*\sigma _3. \end{aligned}$$

#### Proof

As shown above, the spinor $\varPsi _H$ (3.47) and the gauge potential $H^*A+ \frac{3}{4} H^*\sigma _3$ satisfy the coupled equations (3.46). By virtue of the nonlinear equation (3.51), the spin density for $\varPsi _H$ automatically has vanishing divergence. Using the expression (3.47) and the modulus-argument decomposition (3.32) pulled back to $\mathbb {R}^3$,i.e.

3.64 $$\begin{aligned} H^*\varPhi = e^{\frac{1}{2}H^*M + i H^*\chi }, \end{aligned}$$

one then computes

3.65 $$\begin{aligned} A_{\varPsi _H}= & {} -\frac{1}{2}\star \left( \hbox {d} (H^*M) \wedge \theta _{3}\right) -\hbox {d} (H^*\chi ) \nonumber \\&-(1,0) \left( \frac{\varOmega ^2}{2} \star \hbox {d} \left( \frac{G^{-1}\hbox {d} \mathbf {x} \cdot \varvec{\tau }G }{\varOmega ^2}\right) -i G^{-1} \hbox {d}G\right) \begin{pmatrix} 1\\ 0 \end{pmatrix}, \end{aligned}$$

where all Hodge star operations now refer to $\mathbb {R}^3$. The first two terms combine to the expression (3.61) for $H^*A$. The first term inside the expectation value can be rewritten, using (2.31) and (2.30):

3.66 $$\begin{aligned} \frac{\varOmega ^2}{2} \star \hbox {d} \left( \frac{G^{-1}\hbox {d} \mathbf {x} \cdot \varvec{\tau }G }{\varOmega ^2}\right)= & {} - \varOmega ^ 2 \star \hbox {d} \left( \frac{i}{\varOmega } H^{-1} \hbox {d}H \right) \nonumber \\= & {} -i H^{-1}\hbox {d}H +i\star ( \hbox {d}\varOmega \wedge H^{-1} \hbox {d}H), \end{aligned}$$

where we used

3.67 $$\begin{aligned} \star \hbox {d}(H^{-1} \hbox {d}H)=\frac{1}{\varOmega }H^{-1} \hbox {d}H. \end{aligned}$$

Next we use the relation (2.34) to express $G^{-1}\hbox {d}G$ in terms of *H* and $\varOmega$, to deduce

3.68 $$\begin{aligned} \frac{\varOmega ^2}{2} \star \hbox {d} \left( \frac{G^{-1}\hbox {d} \mathbf {x} \cdot \varvec{\tau }G }{\varOmega ^2}\right) -i G^{-1} \hbox {d}G = -\frac{3i}{2} H^{-1}\hbox {d}H. \end{aligned}$$

Then, the observation

3.69 $$\begin{aligned} (1,0)\left( \frac{3i}{ 2} H^{-1}\hbox {d}H \right) \begin{pmatrix} 1\\ 0 \end{pmatrix} = \frac{3}{4}H^*\sigma _3 \end{aligned}$$

completes the proof. $\square$

Formula (3.62) is the starting point of several treatments in the literature of magnetic zero-modes, particularly in the papers [2–4] by Adam, Muratori and Nash (AMN). The AMN construction gives magnetic zero-modes in terms of solutions of the Liouville equation. However, by effectively pulling back local expressions for sections on $S^2$ to $S^3$ via the Hopf map it introduces additional singularities which we will discuss in more detail in Sect. 4.2.

### Zero-mode combinatorics

It is natural to wonder if the linear magnetic zero-modes (2.62) and the vortex zero-modes (3.47) can be combined to produce new zero-modes. This is indeed possible when one picks $s=j$ in (2.62), and notes that, according to (2.50),

3.70 $$\begin{aligned} Y^j_{jm} = C_{jmj}z_1^{j-m} z_2^{j+m}, \quad j\in \frac{1}{2} \mathbb {N}^0, \end{aligned}$$

so that a linear combination of such functions gives another homogeneous polynomial

3.71 $$\begin{aligned} P= A_0z_1^{2j}+ A_1z_1^{2j-1}z_2 + \cdots + A_{2j}z_2^{2j}, \end{aligned}$$

of degree 2*j*. Since such a polynomial satisfies $iX_3P= j P$ and $X_+P=0$, it is easy to check that one can combine it with a vortex configuration $(\varPhi ,A)$ of degree $2n-2\ge 0$ to get a solution

3.72 $$\begin{aligned} \varPsi = G\varOmega ^{-1} H^* \begin{pmatrix} P\varPhi \\ 0 \end{pmatrix}, \quad H^*A'= H^*A + \left( j+ \frac{3}{4}\right) H^*\sigma _3, \end{aligned}$$

of the coupled Dirac equations (3.46) on $\mathbb {R}^3$. Physically, the inclusion of *P* in the spinor adds a multiple of the background field $\mathbf {b}$ to the solution.

There is an obvious mirror version of all our solutions in the anti-holomorphic world: for negative *n* and $s=-j$, one can write down vortex configurations $(\bar{\varPhi },-A)$ in terms of anti-holomorphic polynomials, and obtain corresponding Dirac zero-modes

3.73 $$\begin{aligned} \varPsi = G\varOmega ^{-1} H^* \begin{pmatrix} 0 \\ \bar{P} \bar{\varPhi } \end{pmatrix}, \end{aligned}$$

of the Dirac operator coupled to $-H^*A - \left( j+ \frac{3}{4}\right) H^*\sigma _3.$ These are nothing but the charge conjugates (3.54) of the holomorphic solutions (3.72).

## Popov vortices on $S^2$ and Cartan connections

### Popov vortices from vortices on $S^3$

We now turn to the promised explanation of the link between our vortex equations on $S^3$ and vortex equations on $S^2$ whose solutions are called Popov vortices. Before we write down the equations, we introduce our notation for the round geometry of the 2-sphere.

In a stereographic coordinate *z* defined by projection from the south pole, the round metric of a 2-sphere of arbitrary radius $\lambda$ is

4.1 $$\begin{aligned} \hbox {d}s^2 = \frac{4\lambda ^2 \hbox {d}z \hbox {d}\bar{z}}{(1+|z|^2)^2}, \end{aligned}$$

and a possible complexified frame field $e=e_1+ie_2$ is given by

4.2 $$\begin{aligned} e=\frac{2\lambda }{1+|z|^{2}}\hbox {d}z,\quad \bar{e}=\frac{2\lambda }{1+|z|^{2}}\hbox {d}\bar{z}. \end{aligned}$$

In terms of this frame field, the structure equations can be written as a single complex equation

4.3 $$\begin{aligned} \hbox {d}e-i\Gamma \wedge e =0, \end{aligned}$$

which determine the spin connection 1-form $\Gamma$ as

4.4 $$\begin{aligned} \Gamma = i\frac{z\hbox {d} \bar{z} -\bar{z} \hbox {d}z}{1+|z|^2}. \end{aligned}$$

The topology of the 2-sphere does not permit a globally defined frame, and one checks that the frame (4.2) is singular at $z=\infty$ (the south pole) by switching to $\zeta =1/z$ [11] and noting that *e* behaves likes $\bar{\zeta }^2/|\zeta |^2 \hbox {d}\zeta$ near $\zeta =0$; $\Gamma$, too, has a singularity at $z=\infty$. In our chart, the Riemann curvature form is

4.5 $$\begin{aligned} {\mathcal {R}} = \hbox {d}\Gamma , \end{aligned}$$

and is related to the frame via the usual Gauss equation

4.6 $$\begin{aligned} {\mathcal {R}} = K e_1\wedge e_2 = \frac{i}{2\lambda ^2}e\wedge \bar{e} , \end{aligned}$$

where $K=1/\lambda ^2$ is the Gauss curvature. Thus,

4.7 $$\begin{aligned} {\mathcal {R}} = 2i\frac{\hbox {d}z \wedge \hbox {d}\bar{z} }{(1+|z|^2)^2}, \end{aligned}$$

which integrates to $4\pi$.

In [9], $\lambda =\sqrt{2}$ is chosen and the Popov equations are expressed in terms of the associated Kähler form. However, as we shall see it is more natural to write them in terms of the Riemann curvature form. A Popov vortex is defined on a principal *U*(1) bundle of degree $2n-2$ over the 2-sphere. It is a pair $(\phi ,a)$ of a connection *a* on this bundle and a section $\phi$ of the associated complex line bundle. With $a= a_z \hbox {d}z + a_{\bar{z} }\hbox {d}\bar{z}$ and $f=f_{z\bar{z}} \hbox {d}z\wedge \hbox {d}\bar{z}=da$, the vortex equations in [9] are

4.8 $$\begin{aligned} \partial _{\bar{z}} \phi -i a_{\bar{z}} \phi =0, \quad f= (|\phi |^2 -1) {\mathcal {R}}. \end{aligned}$$

As also explained in [9], solutions are obtained from rational maps $R: S^2 \rightarrow S^2$ of degree *n* which, in our coordinate *z*, take the form (3.25). The Popov vortices are determined by

4.9 $$\begin{aligned} a= R^*\Gamma -\Gamma , \quad R^*e= \phi e. \end{aligned}$$

The second of these equations determines $\phi$ as

4.10 $$\begin{aligned} \phi = \frac{R' (1+|z|^2)}{1+|R|^2} = (p_2' p_1- p_1' p_2)\frac{1+|z|^2}{|p_1|^2 + |p_2|^2}\frac{\bar{p}_1}{p_1}, \end{aligned}$$

which has singularities at the zeros of $p_1$ which we will discuss below (see also [9]). Note also that (4.9) implies that

4.11 $$\begin{aligned} R^*(e\wedge \bar{e})= |\phi |^2 e\wedge \bar{e}, \end{aligned}$$

so that

4.12 $$\begin{aligned} f= \hbox {d}(R^*\Gamma -\Gamma ) = R^*{\mathcal {R}} - {\mathcal {R}} = (|\phi |^2 -1) {\mathcal {R}}. \end{aligned}$$

follows immediately.

We would like to relate the Popov equations and their solutions to vortices on the 3-sphere studied in Sect. 3.1. As reviewed in Sect. 2.1 the Hopf projection $S^3\simeq SU(2)\rightarrow S^2$ in complex coordinates $(z_1,z_2)$ for $h\in SU(2)$ (2.4) and the complex stereographic coordinate *z* on $S^2$ is $\pi : h \mapsto z_2/z_1$, and a local section of this bundle is given by (2.16).

#### Lemma 4.1

The pull-back of the vortex equations (3.3) on $S^3$ via the section *s* (2.16) yields the Popov equations (4.8) up to a singular gauge transformation.

#### Proof

With $U: SU(2) \rightarrow SU(2)$ defined in terms of polynomials $P_1,P_2$ as in (3.8) we define $R:S^2 \rightarrow S^2$ in our stereographic chart by

4.13 $$\begin{aligned} R= \pi \circ U \circ s. \end{aligned}$$

This is the rational map already encountered in the proof of Theorem 3.2, see also the diagram (3.18); it is of the form (3.25). One checks that

4.14 $$\begin{aligned} \pi ^*\Gamma = -\sigma _3+ i \hbox {d}\ln \frac{z_1}{\bar{z}_1}, \end{aligned}$$

so that the pull-back with (2.16) gives

4.15 $$\begin{aligned} s^*\sigma _3 =-\Gamma . \end{aligned}$$

Pulling back (4.14) further with *U*

4.16 $$\begin{aligned} U^*\pi ^*\Gamma = -U^*\sigma _3+ i \hbox {d}\ln \frac{P_1}{\bar{P}_1} \end{aligned}$$

and then with *s*

4.17 $$\begin{aligned} s^*U^*\pi ^*\Gamma = -s^*U^*\sigma _3+ i \hbox {d}\ln \frac{p_1}{\bar{p}_1} \end{aligned}$$

we deduce from the definition of *R* that

4.18 $$\begin{aligned} R^*\Gamma = -s^*U^*\sigma _3+ i \hbox {d}\ln \frac{p_1}{\bar{p}_1}. \end{aligned}$$

Thus, we find that the pull-back of the 1-form $A=U^*\sigma _3-\sigma _3$ is

4.19 $$\begin{aligned} s^*A = s^*U^*\sigma _3 - s^*\sigma _3 = -R^*\Gamma + \Gamma + i \hbox {d}\ln \frac{p_1}{\bar{p}_1}. \end{aligned}$$

Also noting

4.20 $$\begin{aligned} s^*\varPhi = \frac{p_1}{{\bar{p}}_1}\phi , \quad s^*\sigma = \frac{i}{\lambda }e , \end{aligned}$$

and combining this to pull-back the vortex equations on $S^3$ (3.3), we obtain

4.21 $$\begin{aligned}&(\hbox {d}(s^*\varPhi ) +i (s^*A)(s^*\varPhi ))\wedge e = 0, \nonumber \\&\quad \hbox {d}s^*A = \frac{i}{2\lambda ^2} (|\phi |^2-1)\bar{e} \wedge e , \end{aligned}$$

or, equivalently,

4.22 $$\begin{aligned}&\left( \hbox {d}\left( \frac{p_1}{{\bar{p}}_1}\phi \right) +i \left( -R^*\Gamma + \Gamma + i \hbox {d}\ln \frac{p_1}{\bar{p}_1}\right) \left( \frac{p_1}{{\bar{p}}_1}\phi \right) \right) \wedge e = 0, \nonumber \\&\quad -\hbox {d}(R^*\Gamma ) + \hbox {d}\Gamma = -(|\phi |^2-1){\mathcal {R}} . \end{aligned}$$

Writing this in terms of the Popov connection *a*, we conclude that

4.23 $$\begin{aligned}&\left( \hbox {d} \phi -ia \phi \right) \wedge e = 0, \nonumber \\&\quad -f = -(|\phi |^2-1){\mathcal {R}}, \end{aligned}$$

which is equivalent to the Popov equations (4.8). $\square$

### Geometrical interpretation and singularities

It is implicit in our summary, particularly in Eq. (4.9), that Popov vortices can also be interpreted purely geometrically. A metric viewpoint was emphasised and discussed in the more general context of vortex equation on a Riemann surface with a Kähler metric in [10]. In that paper, Baptista pointed out that vortices on a surface with metric *g* define a new geometry by rescaling with the Higgs field

4.24 $$\begin{aligned} g\rightarrow g'=|\phi |^2 g. \end{aligned}$$

The new metric degenerates precisely at the zeros $Z_j$, $j=1,\dots ,2n-2$ of the Higgs field (not necessarily distinct), but its Levi–Civita connection has a Riemann curvature 2-form which, as explained in [10], can naturally be extended to the zeros by including delta function singularities

4.25 $$\begin{aligned} \mathcal {R}'= \mathcal {R} + f -2\pi \sum _{j=1}^{2n-2} \delta _{Z_j}. \end{aligned}$$

Geometrically, the rescaled metric $g'$ has a singularity with a surplus angle $2\pi n$ at a zero of multiplicity *n*. Such singularities can also be thought of as conical singularities with a ‘negative deficit’ angle, i.e. with an excess angle. They resemble a ruffled collar, and are sometimes called ‘Elizabethan geometries’ in the literature.

By virtue of *a* being a connection on a line bundle of degree $2n-2$, we know that

4.26 $$\begin{aligned} \int _{S^2} f = 4\pi n - 4\pi . \end{aligned}$$

When integrating $\mathcal {R}'$ this is cancelled by the delta function contributions, and so

4.27 $$\begin{aligned} \int _{S^2} \mathcal {R}' = 4\pi , \end{aligned}$$

assuring that the usual Gauss–Bonnet formula applies to $\mathcal {R}'$. This should be contrasted with the pull-back curvature $R^*\mathcal {R}$ which integrates to $4\pi n$.

In the metric interpretation, the zeros of the Higgs field lead to singularities, whereas the actual singularities of the Higgs field do not appear to play a special role. To understand the geometric interpretation of the singularities of the Higgs field, we need to consider the frame field defined by it. The complexified frame field

4.28 $$\begin{aligned} \phi e = 2 \lambda \frac{p_2' p_1- p_1' p_2}{|p_1|^2 + |p_2|^2}\frac{\bar{p}_1}{p_1} \, \hbox {d}z \end{aligned}$$

has singularities at each the zeros of $p_1$, i.e. at each of the pre-images of the singularity of the frame *e* under the map *R*. If *q* is a zero of $p_1$, the behaviour near *q* is

4.29 $$\begin{aligned} \phi e \sim A \frac{\bar{z}-\bar{q}}{z-q}\hbox {d}z, \end{aligned}$$

for some constant *A*. Near $z=\infty$, we use again $\zeta =1/ z$ to write the leading term as

4.30 $$\begin{aligned} \phi e \sim B \frac{\hbox {d}z}{z^2} = -B \hbox {d}\zeta , \quad B \; \text {constant}, \end{aligned}$$

which is smooth. It follows that the winding number of the frame field is localised at the zeros of $p_1$, with each zero (counted with multiplicity) contributing a winding of $4\pi$.

This interpretation is gauge dependent. Using (4.20), we find that the frame field

4.31 $$\begin{aligned} s^*(\varPhi \sigma ) = 2i \left( \frac{p_2' p_1- p_1' p_2}{|p_1|^2 + |p_2|^2}\right) \, \hbox {d}z \end{aligned}$$

has no singularities for finite *z*, but has a singularity at $z=\infty$, where it behaves like

4.32 $$\begin{aligned} s^*(\varPhi \sigma )\sim C \left( \frac{z}{|z|}\right) ^{2n} \frac{\hbox {d}z}{z^2}= - C\left( \frac{\bar{\zeta }}{|\zeta |}\right) ^{2n} \hbox {d}\zeta \end{aligned}$$

for yet another constant *C*. In this gauge, the full phase rotation of $4\pi n$ is concentrated at $z=\infty$.

Our discussion shows that any description of the magnetic zero-modes in terms of the Popov vortex fields invariably has singularities since the Popov vortex is a section of and a connection on a non-trivial bundle, neither of which permits a globally smooth expression. This also applies to the expressions derived in [3, 4], which, in our terminology, express the magnetic zero-modes in terms of the modulus and phase of a scalar Popov vortex field (whose modulus obeys a Liouville equation). While one can shift the location of the singularities with gauge transformations, one cannot remove them on $S^2$.

### Gauge potentials for Cartan connections

Cartan connections combine the frame and spin connection into a non-abelian connection. We now show how the results of the previous section can be expressed in the language of Cartan geometry. We first exhibit a local gauge potential for a Cartan connection constructed from the frame and connection defined by a Popov vortex, and then show how it is related to the gauge potential $\mathcal {A}$ used for describing vortices on the 3-sphere in Theorem 3.2.

#### Lemma 4.2

Combine the frame (4.2) and spin connection (4.4) of the 2-sphere into the *su*(2) gauge potential

4.33 $$\begin{aligned} \hat{A}=-\Gamma t_{3}+\frac{i}{2\lambda }e \,t_{-}-\frac{i}{2\lambda }\bar{e}\,t_{+} \end{aligned}$$

defined on the 2-sphere without the south pole. Then, the flatness condition for $\hat{A}$ is equivalent to the structure equation (4.3) and Gauss equation (4.6) on a 2-sphere of radius $\lambda$. Moreover, the flatness of the pull-back $R^{*}\hat{A}$ via the rational map *R* (3.25) is equivalent to the Popov equations being satisfied by the pair $(\phi ,a)$ defined via (4.9).

In the language of Cartan connections, this lemma says that $\hat{A}$ is a gauge potential for a Cartan connection describing the round 2-sphere and that $R^{*}\hat{A}$ is a gauge potential for a Cartan connection describing the deformed geometry defined by the vortex $(\phi ,a)$.

#### Proof

Calculating the curvature of the connection $\hat{A}$ gives

4.34 $$\begin{aligned} F_{\hat{A}}=\hbox {d}\hat{A}+\frac{1}{2}[\hat{A},\hat{A}]=-(\mathcal {R} -\frac{i}{2\lambda ^{2}}e\wedge \bar{e})t_{3}+\frac{i}{2\lambda } (de-i\Gamma \wedge e) t_- -\frac{i}{2\lambda } (\hbox {d}\bar{e}+i\Gamma \wedge \bar{e})t_+, \end{aligned}$$

from which we can read off that the vanishing of the coefficient of $t_{3}$ is equivalent to the Gauss equation and the coefficients of $t_{\pm }$ vanishing are equivalent to the structure equations. Using (4.9), we have

4.35 $$\begin{aligned} R^{*}\hat{A}=-(a+\Gamma )t_{3}+\frac{i}{2\lambda } \phi e \,t_{-}-\frac{i}{2 \lambda }\bar{\phi }\bar{e}\, t_{+}, \end{aligned}$$

with curvature

4.36 $$\begin{aligned} R^* F_{\hat{A}} =-(\hbox {d}a-(|\phi |^{2}-1)\mathcal {R})t_{3}+\frac{i}{2\lambda } (\hbox {d}\phi -ia\phi )\wedge e \,t_- -\frac{i}{2\lambda }(\hbox {d}\bar{\phi }+ia\bar{\phi })\wedge \bar{e}\,t_+. \end{aligned}$$

This being zero is equivalent to the Popov equations (4.8) being satisfied.$\square$

The gauge potential $R^*\hat{A}$ inherits singularities from the singularities of $\phi e$ discussed earlier. In order to treat this more carefully, we use the notion of a principal divisor $D=\sum n_j q_j$ of degree *n* on $S^2$. Given such a divisor we construct a bundle over $S^2{\setminus } \{q_j\}$ by removing the union of the fibres over the $q_j$ from *SU*(2), obtaining the total space

4.37 $$\begin{aligned} P_D= SU(2) {\setminus } \bigcup _j \pi ^{-1} (q_j). \end{aligned}$$

For a homogeneous polynomial *P* of degree *n* in $z_1,z_2$, let *D* be the divisor of zeros of the associated inhomogeneous polynomial *p* (so $P(z_1,z_2)= z_1^n p(\frac{z_2}{z_1})$). Then, we can define the map

4.38 $$\begin{aligned} r_{P}:P_D \rightarrow SU(2), \quad r_P = \begin{pmatrix} \frac{\bar{P}}{|P|}&{}\quad 0\\ 0&{}\quad \frac{P}{|P|} \end{pmatrix} \end{aligned}$$

and the pull-back

4.39 $$\begin{aligned} r_p=s^*r_P: S^2 {\setminus } \{q_j\} \rightarrow SU(2). \end{aligned}$$

It has the form

4.40 $$\begin{aligned} r_{p}=\begin{pmatrix} \frac{\bar{p}}{|p|}&{}\quad 0\\ 0&{}\quad \frac{p}{|p|} \end{pmatrix}. \end{aligned}$$

For later use we note the behaviour of this matrix under fibre rotations. Identifying *h* with $(z_{1},z_{2})$ as in (2.4), we have

4.41 $$\begin{aligned} r_{P}(he^{\frac{\gamma }{n} t_{3}})=e^{-\gamma t_{3}}r_{P}(h), \quad \gamma \in \left[ 0,4\pi \right) . \end{aligned}$$

#### Lemma 4.3

With *s* defined as in (2.16), the gauge potential for the Cartan connection of the 2-sphere is trivialised by *s*:

4.42 $$\begin{aligned} \hat{A} = s^{-1} \hbox {d}s. \end{aligned}$$

Moreover, if *U* is the bundle map (3.8) covering the rational map $R=p_2/p_1$, the gauge potential $R^{*}A$ for the deformed Cartan geometry and the pull-back via *s* of $\mathcal {A}=U^{-1}\hbox {d}U$ are related through the singular gauge transformation $r_{p_1}$:

4.43 $$\begin{aligned} R^*\hat{A} =r_{p_1}^{-1}s^*\left( {\mathcal {A}}\right) r_{p_1}+r_{p_1}^{-1}\hbox {d}r_{p_1}. \end{aligned}$$

#### Proof

Formula (4.42) follows by an elementary calculation and comparison with the definition of *e* and $\Gamma$ in terms of *z* in (4.2) and (4.4). With the map $U:S^3\rightarrow S^3$ defined in terms of polynomials $P_1,P_2$ as in (3.8), and the map $R:S^2\rightarrow S^2$ defined as in (4.13), one checks that

4.44 $$\begin{aligned} U\circ s= \frac{1}{\sqrt{|p_1|^2 + |p_2|^2}} \begin{pmatrix} p_1 &{}\quad -\bar{p}_2 \\ p_2 &{}\quad \bar{p}_1 \end{pmatrix}, \end{aligned}$$

and so, choosing the polynomial $P_1$ used in the definition of *U* (3.8),

4.45 $$\begin{aligned} s\circ R= (U\circ s)r_{p_1}. \end{aligned}$$

It follows that

4.46 $$\begin{aligned} (s\circ R)^{-1} \hbox {d}(s\circ R)= r_{p_1}^{-1}s^*\left( U^{-1}\hbox {d}U\right) r_{p_1}+r_{p_1}^{-1}\hbox {d}r_{p_1}. \end{aligned}$$

Since $${\mathcal {A}}= U^{-1} \hbox {d}U$$ and

4.47 $$\begin{aligned} R^*\hat{A} = (s\circ R)^{-1} \hbox {d}(s\circ R), \end{aligned}$$

the claim follows. $\square$

While the 1-form ${\mathcal {A}}= U^{-1} \hbox {d}U$ is manifestly smooth on $S^3$, its pull-back with *s* is not. The map $s\circ U$ (4.44) has a singularity of the form $z^n/|z|^n$ at $z=\infty$, as one would expect since the pull-backs $s^*P_1$ and $s^*P_2$ are local expression for sections of line bundles of degree *n* over $S^2$ [11]. It follows that the pull-back $s^* {\mathcal {A}}$ is singular at $z=\infty$, with the singularity already exhibited at (4.32).

### Cartan geometry

Our description of the geometry of the 2-sphere and its pull-back via the rational map *R* in terms of *su*(2) gauge potentials has been entirely local so far. It is time to address the global geometrical structure behind these gauge potentials. We will specify the bundles and the connections for which (4.33) and (4.35) are local gauge potentials in the language of Cartan geometry, but refer the reader to the textbook [16] and particularly to the PhD thesis [17] for general definitions and facts about Cartan geometry.

Cartan connections describe the geometry of manifolds modelled on homogeneous spaces *G* / *H* in terms of a connection on a principal *G*-bundle *Q* over this manifold. In order to recover the geometry of a manifold from a Cartan connection one needs an additional structure, namely a section of an associated *G* / *H* bundle which is transverse to the connection or, equivalently (as explained in [17]), a principal *H* subbundle *P* of *Q* which is transverse to the connection.

Here, we are interested in the case $G=SU(2),H=U(1)$ and $G/H=S^2$ and only consider flat Cartan connections. However, we will need to extend the usual framework of Cartan connections to deal with singularities. Consider a divisor *D* of degree *n* on $S^2$, and define the quotient

4.48 $$\begin{aligned} P_{D,n}= P_D/\mathbb {Z}_n, \end{aligned}$$

where $P_D$ is as defined in (4.37) and we think of $\mathbb {Z}_n$ as the subgroup generated by $e^{\frac{4\pi }{n} t_3}$, acting from the right on *SU*(2). This is a *U*(1) bundle over $S^2{\setminus }\{q_j\}$ with the projection provided by the usual Hopf map $\pi$ (2.15). It is a Lens space with *n* circles removed.

In order to construct the required principal *SU*(2) bundle, we define the *SU*(2)-bundle associated with $P_D$ via a *U*(1) action on *SU*(2):

4.49 $$\begin{aligned} Q_D= \{ (h,g) \in P_D \times SU(2)\}/\sim , \end{aligned}$$

where $\sim$ is the equivalence relation

4.50 $$\begin{aligned} (h,g) \sim \left( he^{\frac{\gamma }{n} t_3}, g e^{\gamma t_3}\right) , \quad \gamma \in [0,4\pi ). \end{aligned}$$

This is an *SU*(2) bundle over $S^2{\setminus } \{q_j\}$ with projection

4.51 $$\begin{aligned} \Pi : Q_D\rightarrow S^2 {\setminus } \{q_j\}, \quad \Pi ((h,g)) = \pi (h). \end{aligned}$$

To make this into a principal *SU*(2) bundle, we pick a homogeneous polynomial *P* of degree *n* and consider the map $r_P$ in (4.38). Then,

4.52 $$\begin{aligned} {\tilde{g}} =g r_P \end{aligned}$$

is well defined on $Q_D$, by (4.41), and we define the *SU*(2) right action as

4.53 $$\begin{aligned} u: (h,g) \mapsto (h,gr_P u). \end{aligned}$$

Sections are constructed from maps satisfying an equivariance condition

4.54 $$\begin{aligned} U:P_D\rightarrow SU(2), \quad U\left( he^{\frac{\gamma }{n} t_3}\right) =U(h)\left( e^{\gamma t_3}\right) , \quad \gamma \in [0,4\pi ). \end{aligned}$$

This ensures that, for any *U* satisfying this condition,

4.55 $$\begin{aligned} S_U:S^2{\setminus } \{q_j\} \rightarrow Q_D, \quad n \mapsto (h, U(h)), \quad \pi (h) =n, \end{aligned}$$

is a well-defined section.

The Maurer–Cartan form $g^{-1} \hbox {d}g$ is not well defined on $Q_D$ since it is not right-invariant. However, for any *P*, the 1-form

4.56 $$\begin{aligned} \omega = {\tilde{g}}^{-1} \hbox {d}{\tilde{g}} \end{aligned}$$

is well defined and satisfies the equivariance condition for a connection 1-form. It is the Cartan connection which we are looking for. The map *U* in (3.8) satisfies (4.54). Picking $P=P_1$ and pulling back $\omega$ to our stereographic coordinate chart via $S_U$ lead to the gauge potential

4.57 $$\begin{aligned} (S_U)^*\omega = s^*( (U r_{P_1})^{-1} \hbox {d}(U r_{P_1}) = (s\circ R)^{-1} \hbox {d}(s\circ R), \end{aligned}$$

which, according to (4.47) and (4.35), indeed captures the geometry induced by the Popov vortices.

To end this section, we also exhibit the second way in which one recovers geometry from a Cartan connection. As mentioned earlier, this requires a transverse section of the $SU(2)/U(1) \simeq S^2$ bundle associated with the principal *SU*(2) bundle $Q_D$. In the trivialisation via (4.55), this section (often called the Higgs field in the physics literature on Cartan connections) is simply the constant map

4.58 $$\begin{aligned} \varphi : \mathbb {C}\rightarrow S^2, \quad z\mapsto t_3, \end{aligned}$$

where we think of $t_3$ as an element of unit sphere inside *su*(2). The geometry is recovered from the covariant derivative

4.59 $$\begin{aligned} D_{R^*\hat{A}} \varphi = [R^*\hat{A}, t_3], \end{aligned}$$

which extracts the frame $\phi e$ from the gauge potential $R^*\hat{A}$. If we apply the gauge transformation $s^*(Ur_{P_1})$, the gauge potential vanishes in the new gauge, but the transverse section is now

4.60 $$\begin{aligned} {\tilde{\varphi }} : z\mapsto Ut_3U^{-1}, \end{aligned}$$

which, up to stereographic projection, is our rational map *R*. In other words, the rational map which solves the Popov vortex equations is the ‘transverse Higgs field’ of Cartan geometry in a particular gauge. The geometry is still recovered via the covariant derivative, but since the gauge potential vanishes now, this is simply the exterior derivative $d \tilde{\varphi }$ which, modulo stereographic projection, indeed reproduces the formula for the frame $\phi e$ in terms of the derivative of *R*.

## Summary and outlook

**Fig. 3:**
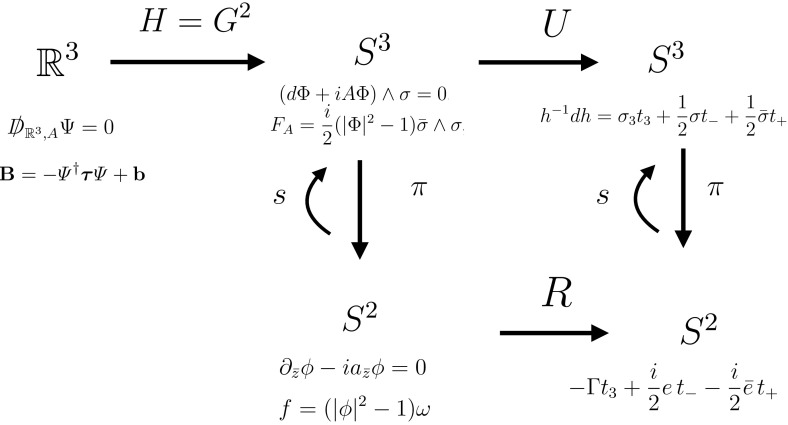
A summary of the equations and maps studied in this paper

The equations, spaces and maps studied in this paper are summarised in Fig. 3, with magnetic zero-modes on the top left of the picture and the Popov vortex equations on the bottom. The geometry of the 3-sphere, as encoded in the Maurer–Cartan form $h^{-1}\hbox {d}h$, and its pull-back via the bundle map *U* provides a unifying point of view for both and leads to the explicit and smooth description of both vortex zero-modes and of vortices.

We believe that our results provide a fully geometrical understanding of magnetic zero-modes on $\mathbb {R}^3$ and would like to stress the practical advantage of having manifestly singularity-free and square-integrable formulae. Previous expressions based on the Loss–Yau formula (3.62) have singularities where the spinor field vanishes, see also our discussion at the end of Sect. 4.2.

The diagram in Fig. 3 shows that the structures studied in this paper are closely related to many of the most studied topological solitons [18]. Apart from the obvious vortex configurations, the maps *U* and their pull-backs $U\circ H$ are topologically Skyrme fields on $S^3$ and $\mathbb {R}^3$. The rational maps *R* can also be interpreted as ‘lumps’ or baby Skyrmions on the 2-sphere, while the composite maps $H\circ U\circ \pi = H\circ \pi \circ R$ are topologically Hopfions. As discussed in Sect. 3.2, the magnetic fields on $\mathbb {R}^3$ obtained by pulling back the area form on the unit 2-sphere via these maps are examples of linked magnetic fields of the kind studied by Rañada and more recently, in more detail and with many pictures, in [14]. Finally, the equation obeyed by vortex zero-modes on $\mathbb {R}^3$ is related to the Seiberg–Witten equations on $\mathbb {R}^4$ with a sign flipped [2].

It is clear that much of what we discussed in this paper has a close analogue in a Lorentzian and hyperbolic setting, where the 2-sphere is replaced by hyperbolic 2-space, the 3-sphere by 3-dimensional anti-de Sitter space (or *SU*(2) by *SU*(1, 1)), and Euclidean $\mathbb {R}^3$ by Minkowski space $\mathbb {R}^{2,1}$. Spelling this out is the topic of a forthcoming paper [19]. However, one should also consider further generalisations suggested by the origin of the Popov equations in self-duality.

As we briefly mentioned in the Introduction, the Popov equations are symmetry reduction of the self-duality equations for *SU*(1, 1) instantons on $\mathbb {R}^4$. In fact, there is a whole family of integrable vortex equations recently studied by Manton [20] which have similar links to self-duality equations, with the relevant gauge group depending on the vortex equation [21]. It is intriguing that, for Popov vortices, the non-abelian gauge group *SU*(1, 1) needed for the self-dual connection differs from the *SU*(2) we used in our description in terms of flat Cartan connections. It would be interesting to understand both viewpoints and their relationship systematically for the family of integrable vortex equations studied in [20].

To end, we point out that interactions of spinors with linked magnetic fields of the form (3.56) are currently much discussed in atomic and condensed matter physics, where the spinors arise as an effective description of nearly degenerate states of ultra-cold atomic Bose-Einstein condensates, and the magnetic field as the curvature of a Berry connection, see, for example, the papers [22, 23] for a review. Our explicit expression for more general magnetic zero-modes may prove useful in that context.
